# Synthesis and anti-obesity effects *in vivo* of Crotadihydrofuran C as a novel PPARγ antagonist from *Crotalaria albida*

**DOI:** 10.1038/srep46735

**Published:** 2017-04-24

**Authors:** Qin-Hu Sun, Yu Zhang, Gui-Xin Chou

**Affiliations:** 1 The MOE Key Laboratory for Standardization of Chinese Medicines and SATCM Key Laboratory for New Resources and Quality Evaluation of Chinese Medicines, Institute of Chinese Materia Medica of Shanghai University of Traditional Chinese Medicine, Cai Lun Road 1200, Zhangjiang, Shanghai, 201210, People’s Republic of China; 2 Shanghai R&D Center for Standardization of Chinese Medicines, Shanghai 201203, People’s Republic of China; 3 School of Life Science and Technology, ShanghaiTech University, Shanghai 201210, China

## Abstract

Crotadihydrofuran C (CC) from the herbs of Crotalaria albida is able to inhibit adipocyte differentiation and lipid accumulation. However, the effects of CC on obesity and metabolic disorders have not yet been elucidated. In our study, the first enantioselective synthesis of the 2-isopropenyl dihydrofuran isoflavone skeleton (CC) is described. The convenient and efficient synthetic protocols developed skilfully solve the problems of the ortho-para directing group and Suzuki coupling reaction using a boronic acid pinacol ester that was more stable and easy to obtain. Furthermore, CC treatment of high-fat diet (HFD)-fed obese mice remarkably reduced their body weight, fat mass, and lipid level as well as improved insulin resistance and non-alcoholic fatty liver disease (NAFLD). A TR-FRET assay showed that CC was specifically bound to PPARγ LBD, which was further confirmed by the molecular docking study. These results suggest that CC could be a useful and potential natural product for treating metabolic diseases, including obesity, hyperlipidemia insulin resistance and NAFLD, without toxic side-effects.

Obesity is caused by an imbalance between energy expenditure and food consumption and is frequently associated with the development of serious chronic diseases such as atherosclerosis, hypertension, insulin resistance, hyperlipidemia and fatty liver^1^,^2^,^3^,^4^. It has emerged as one of the top public health problems, and the number of overweight and obese individuals is expected to be over half of the world’s population by 2030^5^. Obesity is characterized by both increased adipocyte size (hypertrophy) and adipocyte number (hyperplasia). Thus, lipogenesis is a key process that controls the development of obesity^6^,^7^.

The nuclear receptor peroxisome proliferator-activated receptor γ (PPARγ) is a ligand-activated transcription factor, which is involved in lipogenesis as well as glucose and energy homeostasis, and it has been identified as a therapeutic target for metabolic diseases^8^,^9^,^10^. Thiazolidinediones (TZDs), such as pioglitazone and rosiglitazone, which are classic PPARγ agonists, exert an antidiabetic effect through improving insulin sensitivity, whereas they may cause weight gain and fatty liver disease in patients and animals^11^,^12^,^13^,^14^. In contrast, recent studies have highlighted that PPARγ antagonists ameliorated high-fat diet (HFD)-induced obesity, insulin resistance and fatty liver disease by inhibiting lipogenesis^15^,^16^,^17^,^18^,^19^,^20^,^21^. These results indicate that the inhibition of PPARγ activity could be beneficial to prevent and treat obesity and obesity-related metabolic diseases, and it may even be superior to activation in terms of obesity based on fat formation and lipogenesis. Numerous pharmacological approaches have been used clinically for the prevention and treatment of obesity. However, the administration of drugs including orlistat, which is a lipase inhibitor, led to undesirable side-effects, such as insomnia, constipation, headaches and heart attacks^22^,^23^,^24^. Therefore, it is desirable to develop safe and effective therapeutic drugs to treat diet-induced obesity and other metabolic disorders.

Natural products could be a source for the development of potential therapeutic drugs for metabolic disorders. However, studies cannot be carried out *in vivo* due to limited amounts of the natural products. In our previous research, we reported the isolation, structural determination and evaluation of CC. It has been determined that CC is capable of inhibiting 3T3-L1 preadipocyte differentiations by decreasing PPARγ transactivity induced by rosiglitazone^25^. However, the anti-obesity effects *in vivo* are totally unknown. In our study, we designed a series of efficient protocols and achieved the first chiral synthesis of CC in 16 steps. We examined the therapeutic effects of CC on obesity and obesity-associated glucose and lipid disorders, hepatic steatosis, steatohepatitis and fibrosis in HFD obesity models. Moreover, we performed a competitive binding assay to confirm the PPARγ antagonism of CC. Our data indicated that CC treatment could improve obesity, insulin resistance, hyperlipidemia and non-alcoholic fatty liver disease (NAFLD) disease in diet-induced obesity (DIO) mice as a novel PPARγ antagonist.

## Results and Discussion

### Chemistry

**Figure 1:**
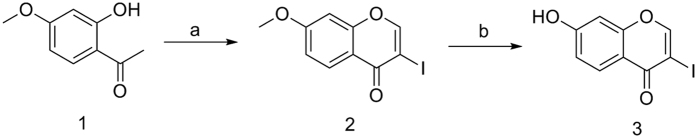
Synthesis of intermediate 3. Reagents and conditions: (**a**) (i) DMF/DMA, 95 °C, 3 h, (ii) TCM, pyridine, I_2_, rt; (**b**) toluene, AlCl_3_, 100 °C.

**Figure 2:**
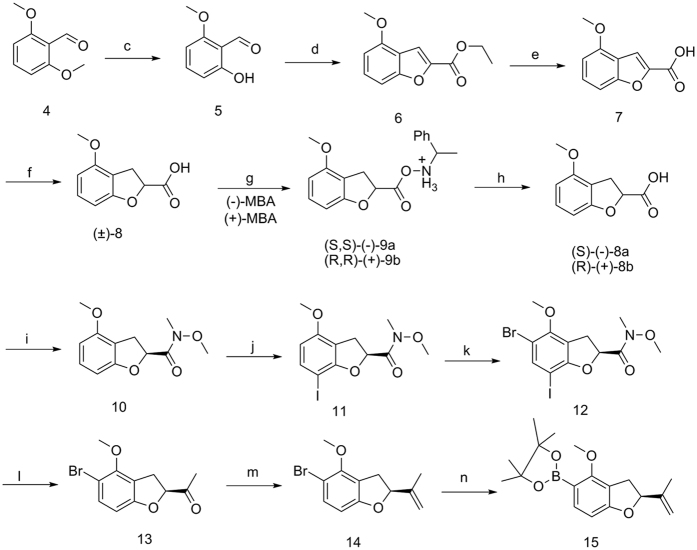
Synthesis of intermediate 15. Reagents and conditions: (**c**) NaI, AlCl_3_, MeCN, DCM, 0 °C; (**d**) BrEA, K_2_CO_3_, DMF, 140 °C; (**e**) MeOH, NaOH, 100 °C; (**f**) MeOH, Mg, rt; (**g**) Me_2_CO, (+)-α-MBA, (-)-α-MBA; (**h**) water, EtOAc, 5 N HCl (aq), 0 °C; (**i**) DCM, CDI, H•HCl, rt; (**j**) TCM, I_2_, silver triflate, rt; (**k**) THF, NBS, rt; (**l**) THF, MeBrMg, −10 °C; (**m**) (i) ether, iodomethane, KTB, 0 °C, (ii) 40 °C; (**n**) 1,4-dioxane, Pd(dppf)Cl_2_, KOAc, bis(pinacolato)diboron, 105 °C.

**Figure 3:**
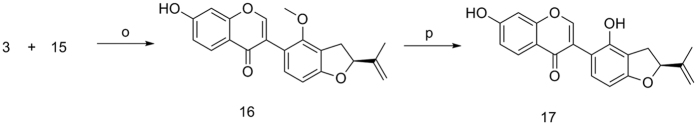
Synthesis of CHF C. Reagents and conditions: (o) 1,4-dioxane, Pd(dppf)Cl_2_, 1 N Na_2_CO_3_ (aq), 50 °C; (p) DMF, sodium ethanethiolate, 100 °C.

The palladium catalysed Suzuki coupling between boronic acid and iodide was considered a major solution in the synthesis of isoflavones, which was due to the excellent electrophilic properties of the iodo moiety that was an ideal substrate due to its unique position (nearest to the double bond). The optimum conditions that used inexpensive and easily available reagents and stocks, had low toxicity so as to be environmentally benign, were very secure, and were stable were considered. Thus, the intermediate (boronic acid pinacol ester, **15**) that was achieved in Pd(dppf)Cl_2_, KOAc and bis(pinacolato)diboron at 105 °C was more stable and easy to obtain than the boronic acid derivative that was produced at −78 °C. The synthesis of intermediate **3** was achieved from Paeonol obtainedvia commerce, condensation with N,N-dimethylformamide dimethylacetal (DMF-DMA) and cyclization using I_2_ to furnish the corresponding iodobenzopyranone. Subsequent methyl ether cleavage of **2** and the addition of AlCl_3_ in toluene provided compound **3** (Fig. 1).

(*S*)-(-)-2,3-Dihydro-2-isopropenyl-4-methoxybenzofuran-5-boronic acid pinacol ester **15** that was considered a key intermediate in the synthesis of 7, 2′dihydroxy-2′′ S-isopropenyl dihydrofuran [6′′, 7′′ : 3′, 4′]-isoflavone **17** was achieved from 2, 6-dimethoxybenzaldehyde through the following sequence of reactions: selective protection of the 2-hydroxy group of 2, 6-dimethoxybenzaldehyde **4** by a methyl ether to furnish 2-hydroxy-6-methoxybenzaldehyde **5**, and cyclization using ethyl bromoacetate provided intermediate **6**. Compound **7** was prepared by basic hydrolysis using sodium hydroxide. Compound **7** was hydrogenated by the repeated addition of magnesium in methanol to yield 2,3-dihydro-2-carboxy-4-methoxybenzofuran **8**, and this racemic compound was resolved using the commercially available enantiomers of α-methylbenzylamine (α-MBA). In consideration of the ortho-para directing group (methoxy), the Weinreb amide **10** was iodinated and brominated in that order to achieve (*S*)-(-)-2,3-dihydro-2-carboxamide-N-methoxy-N-methyl-4-methoxybenzofuran-5-bromo-7-iodo **12** using iodine and N-bromobutanimide, respectively. The ethanone **13** was obtained by an overdose of methylmagnesium bromide in THF. Furthermore, the isopropenyl **14** converted by a Wittig reaction was used to generate compound **15** (Fig. 2). Finally, methyl ether cleavage of **16** was achieved by adding ethanethiol sodium salt in DMF to provide target compound **17** (Fig. 3).

### Crotadihydrofuran C counters obesity and improves HFD-induced obesity in mice

**Figure 4:**
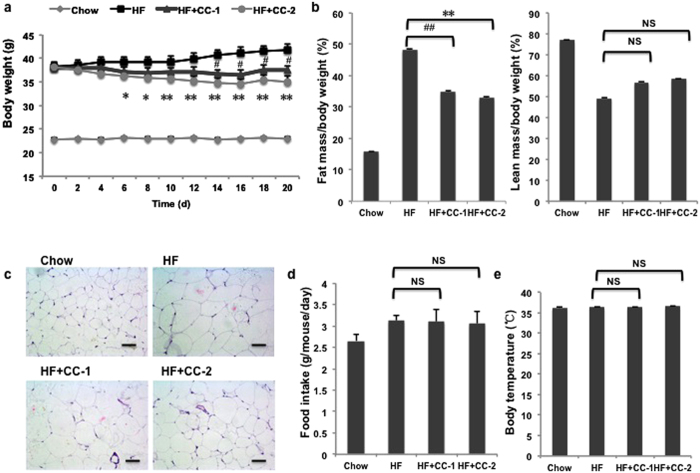
CC treatment reduces body weight and renders mice resistant to diet-induced obesity. (**a**) Body weights of mice were treated with chow diet, high-fat diet or high-fat diet and CC for 20 days (n = 10). (**b**) Percentage of fat and lean mass to body weight ratio. (**c**) Adipocyte size in the WAT of each group (n = 10). WAT was stained with haematoxylin and eosin. Scale bars, 50 μm. (**d**) Daily food consumption (n = 10). (**e**) Body temperature (n = 10). *p < 0.05, **p < 0.01, CC-2 vs HF mice. ^#^p < 0.05, ^##^p < 0.01, CC-1 vs HF mice. NS, no significance. CC-1, 50 mg/kg CC. CC-2, 100 mg/kg CC. HF, high fat. d, day.

Based on the results of our previous studies that CC inhibited PPARγ transactivity and suppressed adipocyte differentiation via reducing mRNA expression of PPARγ target genes *in vitro*^25^, we postulated that CC might have a treatment effect on obesity and metabolic disorders in obese mice. HFD feeding for three months led to a significant increase in the body weight and created an obesity model in C57/BL mice (Fig. 4a). In contrast, supplementation with CC decreased the weight gain and fat accumulation in a dose-dependent manner in HFD-fed mice (Fig. 4a,b). The reduction in weight gain of CC-treated mice was largely attributed to decreased overall fat mass, without any change in the lean mass (Fig. 4b). Histological analysis showed CC treatment caused a remarkable decrease in the size of the adipocytes in the visceral adipose tissue versus mice fed only a high-fat diet (Fig. 4c). In addition, we did not observe obvious clinical signs of toxicity or mortality, such as changes in skin, fur, eyes, gait, posture, response to handling and the bizarre behavior during the entire period of the study, as well as no toxicity to cell proliferation in our previous study. These results revealed that effect of CC on body weight and metabolism could not be caused by CC toxicity. Furthermore, the mean body temperature and food consumption did not vary significantly between the HF and CC groups (Fig. 4d,e), suggesting that the effects of CC on obesity parameters were not due to decreased food intake and energy expenditure. Hence, these data indicated that CC has a beneficial effect in reducing body weight gain and fat accumulation in obese mice.

### CC treatment ameliorates diabetes and hyperlipidemia in DIO mice

**Figure 5:**
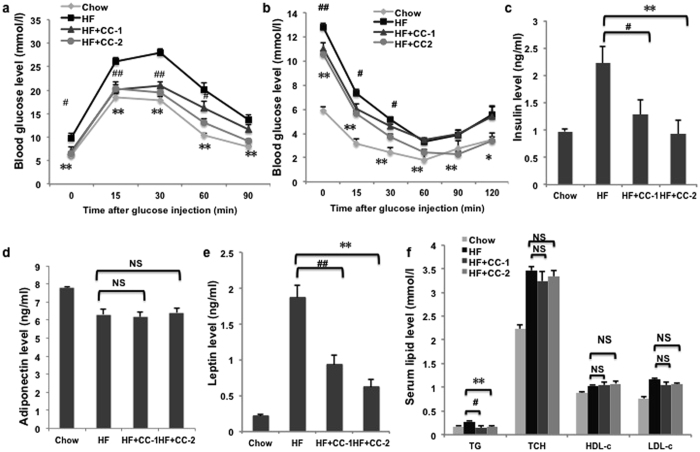
CC administration improves obesity-associated metabolic disorders. (**a**) Glucose levels during ipGTT after 10 h of fasting (n = 10). (**b**) Glucose levels during ipITT without fasting (n = 10). (**c**) Serum insulin, (**d**) adiponectin, (**e**) leptin, (**f**) TG, TC, LDL-c, and HDL-c levels were measured after 10 h of fasting (n = 10). *p < 0.05, **p < 0.01, CC-2 vs HF mice. ^#^*p < *0.05, ^##^*p* < 0.01, NS, no significance.

Consistent with reduced adiposity, CC-treated mice showed significant improvement in their glucose haemostasis(Fig. 5a,b). After 20 days of treatment with CC, mice exhibited remarkable reductions in blood glucose levels following intraperitoneal glucose injection compared with obese control mice, suggesting that CC enhanced glucose tolerance. Similar results were detected in an intraperitoneal insulin tolerance test. Hyperlipidemia is one hallmark of insulin resistance and type 2 diabetes. As shown in Fig. 5c, the serum insulin level of obese mice was higher than that of the low fat diet group. Compared with the model group, an obvious reduction of the insulin level was observed in the CC-treated group. Furthermore, CC remarkably down-regulated the level of leptin, which is the key regulator of body weight secreted by adipocytes, but it did not lead to any change in the adiponectin level (Fig. 5d,e). The above results demonstrated that CC attenuates serum insulin and leptin levels in HFD-induced obese mice, which may bring benefits for the improvement of obesity and insulin resistance.

To investigate the effect of CC on the improvement of lipid metabolism, the levels of total triglyceride (TG), total cholesterol (TC), high-density lipoprotein cholesterol (HDL-c) and low-density lipoprotein cholesterol (LDL-c) were determined in the serum (Fig. 5f). The TG levels of HFD mice increased significantly. After 20 days of treatment, the TG levels of the CC-treated group were effectively reduced and almost reached those of the chow group. However, the TC, HDL-c and LDL-c levels remained unchanged. These results indicated that CC treatment could reduce the serum lipid level and improve hyperlipidemia in DIO mice.

### CC reverses high-fat diet-induced steatosis, steatohepatitis and liver fibrosis in mice

**Figure 6:**
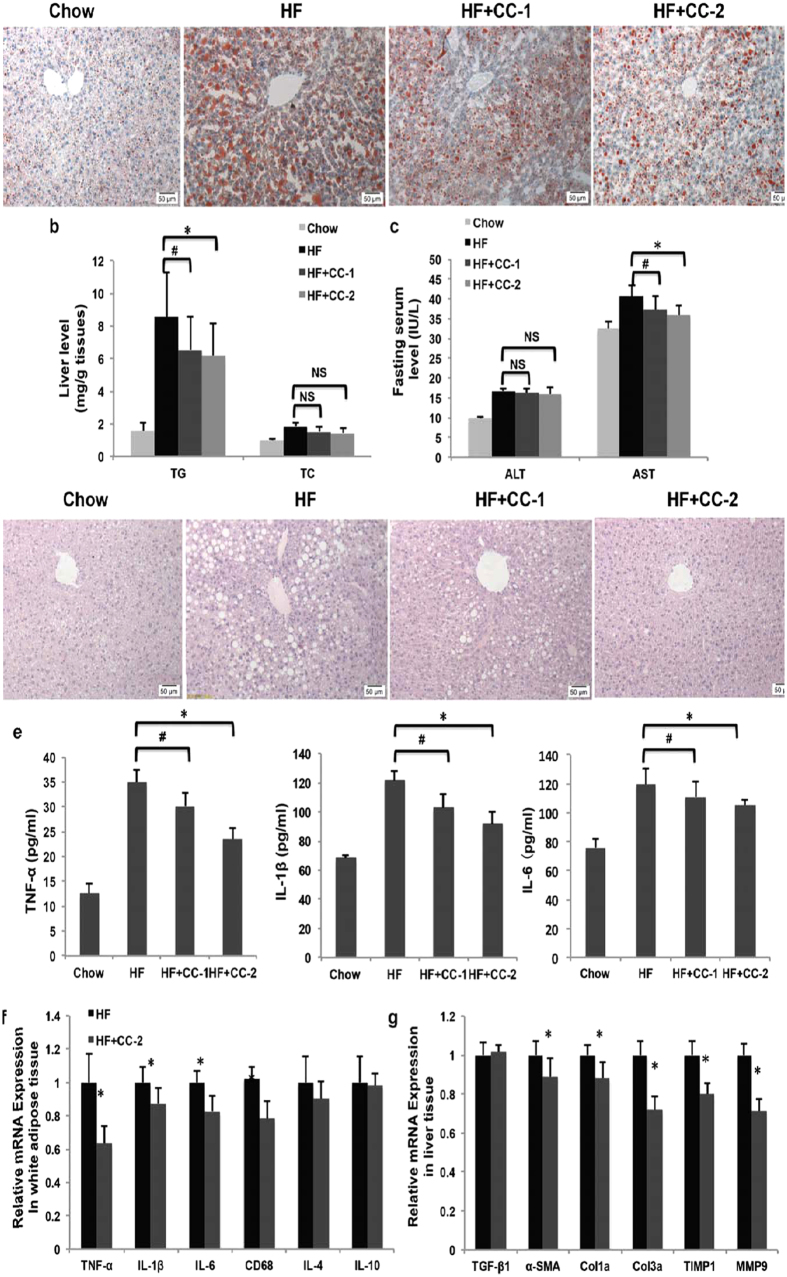
CC alleviated NAFLD in DIO mice after 20 days of treatment. (**a**) Oil Red O staining of liver sections (n = 10). (**b**) Liver TG and TC levels (n = 10). (**c**) Serum ALT and AST levels (n = 10). (**d**) H&E staining of liver sections (n = 10). (**e**) Serum TNF-α, IL-6 and IL-1β levels (n = 10). (**f**) CC reduced mRNA expression of inflammation-related genes in white adipose tissue (n = 6). (**g**) CC reduced mRNA expression of fibrosis-related genes in liver tissue (n = 6). *p < 0.05, **p < 0.01, CC-2 vs HF mice. ^#^*p* < 0.05, ^##^*p* < 0.01, CC-1 vs HF mice. NS, no significance.

NAFLD is frequently associated with insulin resistance and is defined as a liver component of metabolic syndrome, which is related to excessive accumulation of hepatic fat and encompasses a spectrum of conditions ranging from steatosis alone to steatohepatitis with inflammation and fibrosis^26^. To investigate whether CC improves hepaticsteatosis, we compared the profiles of the livers of mice fed a chow diet, HFD alone or HFD containing CC. As shown in Fig. 6a, although HFD induced massive hepatic steatosis, CC decreased lipid accumulation in the liver, which was verified by quantification of the TG content of liver tissues (Fig. 6b). Compared with the HF group, CC lowered the liver TG content by approximately 30% and did not cause any difference in the TC content in obese mice. Next, we determined the contents of alanine aminotransferase (ALT) and aspartate aminotransferase (AST), which are characterized as liver diagnostic markers. The results showed that CC reduced the increase in the plasma concentration of AST induced by HFD. In contrast, CC did not lead to any significant change in the ALT level, but it showed a decreasing tendency (Fig. 6c). Furthermore, histological analyses of the mouse liver demonstrated that CC further ameliorated tissue structure and hepatic steatosis (Fig. 6d). These above results indicated that oral CC is effective in reducing HFD-induced hepatic steatosis in mice.

Heptaic steatosis is a precursor of more advanced liver disease and can progress to non-alcoholic steatohepatitis(NASH), which is characterized by inflammation and fibrosis^27^,^28^,^29^. In order to further confirm that CC adimistration could bring about sustained improvement of liver fibrosis, we next analysed the serum level of pro-inflammatory cytokines such as tumor necrosis factor α (TNF-α), interleukin (IL)-1β and IL-6. As shown in Fig. 6e, the serum level of TNF-α, IL-1β and IL-6 was significantly increased in HF group compared with the chow group. CC treatment remarkably decreased the levels of TNF-α, IL-1β and IL-6. This anti-inflammatory effect was also reflected in reduced the mRNA expression of inflammation mediators including IL-1β, IL-6, TNFα, and cluster of differentiation (CD) 68 in adipose tissue, whereas the levels of IL-4 and IL-10 were not changed remarkably (Fig. 6f). These results suggest that CC may improve inflammatory state in DIO mice. Activation of hepatic stellate cells and extracellular matrix synthesis are responses to hepatic injury^30^. To further determine whether CC protects against HF diet-induced liver fibrosis, we then assessed the hepatic mRNA levels of several marker genes through quantitative PCR (qPCR). As shown in Fig. 6g, expression of transforming growth factor (TGF)-β1, α-smooth muscle actin (SMA), type I collagen (Col1) a and type III collagen (Col3) a significantly increased in mice fed with HF diet. Treatment with CC reversed the increases of α-SMA, Col1a and Col3a expression, which indicate that anti-fibrogenic properties are displayed by CC. In addition to alteration of the expression of pro-fibrogenic genes, we also investigated expression of genes involved in mediating extracellular matrix (ECM) remodelling. Similar to the results of pro-fibrogenic genes, CC administration markedly suppressed unregulated levels of tissue inhibitors of metalloproteinase (TIMP)1, matrix metalloproteinase (MMP) 9 caused by hepatic fat accumulation. Taken together, these results demonstrate that CC effectively reversed HFD-induced hepatic steatosis, inflammation and even fibrosis.

### CC functions as a PPARγ antagonist with a potent binding affinity and regulates PPARγ gene expression *in vivo*

**Figure 7:**
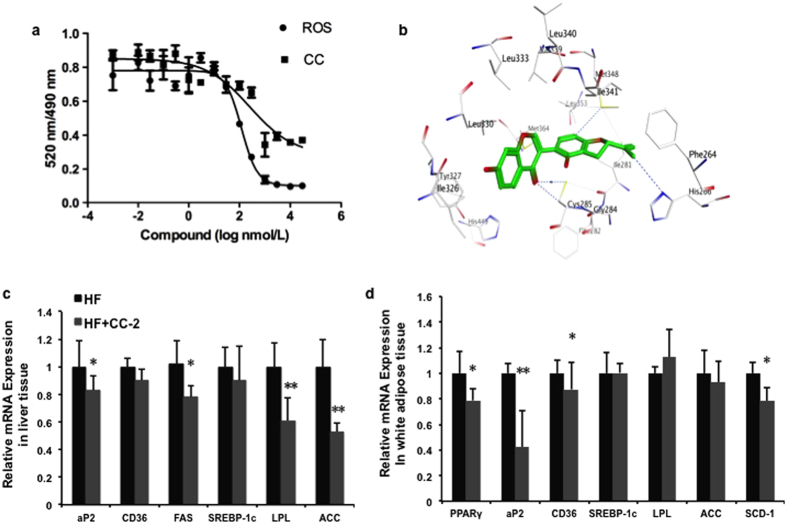
CC functions as a potent PPARγ antagonist and selectively regulates the expression of PPARγ target genes. (**a**) Binding to LBDs of human PPARγ in competition with rosiglitazone in a TR-FRET competitive binding assay (n = 3). (**b**) The structure of the complex of the PPARγ-LBD and CC by molecular docking. (**c**,**d**) CC reduced mRNA expression of PPARγ target genes in liver tissue (**c**) and white adipose tissue (**d**) (n = 6). CC is presented as CC-2 (100 mg/kg) Data are expressed as the mean ± SEM. *p < 0.05, **p < 0.01, CC vs HF mice.

PPARγ is a master regulator of adipocyte differentiation, glucose and lipid metabolism, and inflammation. PPARγ agonists induce lipogenesis and adipocyte differentiation. In contrast, PPARγ antagonists have opposite effects. In our previous studies, several results revealed that CC may be a PPARγ antagonist^25^. First, a reporter assay showed that CC selectively reduced the transactivity of PPARγ induced by rosiglitazone. Second, CC inhibited adipocyte differentiation of 3T3-L1 cells, accompanying with the reduced expression of PPARγ and its downstream genes *in vitro*^25^. To confirm this hypothesis and further confirm the binding of CC to PPARγ, we next performed competitive binding assays using time-resolved fluorescence resonance energy transfer (TR-FRET). As shown in Fig. 7a, CC is capable of dose-dependently displacing the rosiglitazone from binding to PPARγ and showed a strong binding to human PPARγ (K_i_ = 1.57 μM, IC_50_ = 3.65 μM). Next, docking studies were performed to investigate the exact binding sites of CC to PPARγ. Since the CC is in the S configuration, the hydrogen atom of isopropenyl at the 2′ position forms a weak H-bond with His266. The same hydrogen atom could form a H-benzene interaction with Phe264, which enhances the binding stability. In addition, the carbonyl group of chromone forms a strong H-bond with Cys285. Reasonably, we speculate that CC with a S configuration has stronger activity than that of the R configuration. The predicted binding model showed that interaction between CC and the PPARγ ligand binding site is similar to the observation between PPARγ and its known agonist, TZDs. In our present studies, we have confirmed that CC competed with rosiglitazone to bind to the human PPARγ receptor with a Ki of 1.57 μM. Furthermore, molecular docking studies suggested CC may interact with the ligand binding domain of PPARγ, where hydrogen bonds at His266, Phe264 and Cys285 are predicted to be formed. These presented evidences support a notion that CC is a novel PPARγ antagonist.

To reveal the mechanism underlying CC-regulated improvement in metabolic disorders, the gene expression profiles of liver and white adipose tissue were analysed by qPCR. Consistent with the results in 3T3-L1 adipocytes, CC markedly suppressed the expression of the classic PPARγ target gene involved in the insulin sensitivity and synthesis and transport of fatty acids in liver tissue, such as adipose fatty acid-binding protein 2 (aP2), fatty acid synthase (FAS), lipoprotein lipase (LPL) and acetyl-CoA carboxylase (ACC) (Fig. 7c). Similarly, the expressions of PPARγ, aP2, the cluster of differentiation 36 (CD36) and stearoyl-CoA desaturase (SCD) 1 in white adipose tissue were remarkably downregulated in DIO mice (Fig. 7d). These data indicate that CC has been shown to have PPARγ-independent action on fat accumulation and lipogenesis *in vivo*, supporting the idea that CC could be a potential PPARγ antagonist.

In summary, this paper outlines the first high-yielding enantioselective synthesis of the 2-isopropenyl dihydrofuran isoflavone skeleton using a palladium catalyzed Suzuki coupling reaction in the presence of aboronic acid pinacol ester. This is a first chiral synthesis to obtain a large enough enough amount to carry out animal experiments. Furthermore, we identified that CC reduces weight gain and fat accumulation as well as improves glucose homeostasis, hepatic lipid, inflammation and fibrosis as a novel natural antagonist of PPARγ. The potent effect of CC on obesity and metabolic diseases without apparent toxic side-effects makes CC a promising candidate in the development of anti-obesity pharmacotherapy.

## Experimental Section

### General

The 1D and 2D NMR spectra were obtained with Bruker AV-400 or AV-600 instruments at 600\400 MHz for 1 H and 150\100 MHz for 13 C, CD_3_OD (δH 3.33; δ C 49.3). Prep-HPLC (Agilent 1260 Series) was performed on a C-18 column (SHISEID-PACK 20 mm × 250 mm, 5 um). The preparatory silica gels (100−300 mesh) and Sephadex LH-20 were obtained from QMC Co., Ltd. and GE-H Co., Ltd.^31^.

Rosiglitazone was purchased from Enzo Life Science (Lausen, Switzerland). High-fat diet (HFD, Product# D12492) and low-fat diet (LFD Product# D12450B) were obtained from Research Diets (New Brunswick, NJ, USA). HFD with 60 kcal% fat includes 26.2% protein, 26.3% carbohydrate, and 34.9% fat. LFD with 10 kcal% fat includes 19.2% protein, 67.3% carbohydrate and 4.3% fat.

### 7-Methoxy-3-iodochromen-4-one (2)

The Paeonol **1** (10 g, 60 mmol) was diluted with DMF/DMA (15 ml, 90 mmol), and stirred at 95 °C for 3 h to obtained solid. The mixture was dissolved in CHCl_3_ (50 ml) and successively treated with pyridine (4.8 ml, 60 mmol) and I_2_ (30 g, 120 mmol). The resulting mixture was stirred at room temperature for 1 h. The mixture was washed with saturated aqueous Na_2_S_2_O_3_ solution and extracted with CH_2_Cl_2_. The collected organic extracts were dried (Na_2_SO_4_) and concentrated in vacuo. Purification by flash chromatography (20% EtOAc/petroleum ether then 40% EtOAc/petroleum ether) gave **2** (21 g, 90% yield) as a yellow solid^32^; mp 130–135 °C. ^1^H-NMR(400 MHz, CDCl_3_) δ_H_ 8.20 (1H, s), 8.13 (1H, d, J = 8.8 Hz, Ar-H), 6.99 (2H, dd, J = 8.8, 1.6 Hz, Ar-H), 6.82 (1H, s, Ar-H), 3.90(3H, s, OCH_3_); ^13^C-NMR(100 MHz, CDCl_3_) δ_c_ 172.5, 164.3, 157.9, 157.2, 128.0, 115.6, 115.3, 100.0, 87.1, 55.9 (S3 and S4 Figs).

### 7-Hydroxy-3-iodochromen-4-one (3)

To a solution of **2** (10 g, 33 mmol) in toluene (200 ml), AlCl_3_ (6.6 g, 50 mmol) was added with stirring at 100 °C under nitrogen overnight. Then, the reaction residue was poured into 10% HCl (aq) and extracted with EtOAc (200 ml) twice. The organic layers were dried over Na_2_SO_4_ and concentrated in vacuo to dryness. The crude was purified by flash chromatography (20% EtOAc/petroleum ether then 40% EtOAc/petroleum ether) to provide compound **3** (9.1 g, 91% yield); yellow needles^33^; mp 245–250 °C. ^1^H-NMR(600 MHz, CD_3_OD) δ_H_ 8.51 (1H, s), 8.01 (1H, d, J = 8.8 Hz, Ar-H), 6.96 (2H, dd, J = 8.8, 2.2 Hz, Ar-H), 6.85 (1H, d, J = 2.2 Hz, Ar-H); ^13^C-NMR(150 MHz, CD_3_OD)δ_c_ 174.9, 165.0, 160.1, 159.7, 128.7, 117.0, 115.5, 103.1, 86.2 (S5 and S6 Figs).

### 2-Hydroxy-6-methoxybenzaldehyde (5)

To a mixture of **4** (50 g, 300 mmol), NaI (110 g, 734 mmol) in CH_3_CN (700 ml) and CH_2_Cl_2_ (350 ml) at 0 °C AlCl_3_ (100 g, 734 mmol) was added portion-wise over a period of 30 min. The resulting mixture was further stirred at 0 °C for 10 min. TLC showed that the reaction was complete. The mixture was washed with saturated aqueous Na_2_S_2_O_3_ solution, the organic layers were separated and dried over saturated aqueous NaCl, and then, they were concentrated under reduced pressure to give desired products **5** (45.5 g, 90% yield); yellow powder^34^; mp 105–110 °C. ^1^H-NMR(600 MHz, CD_3_OD) δ_H_ 10.3(1H, s), 7.47 (1H, J = 8.4 Hz, Ar-H), 6.53(1H, d, J = 8.3 Hz, Ar-H), 6.48(1H, d, J = 8.4 Hz, Ar-H), 3.83(3H, s, OCH_3_); ^13^C-NMR (150 MHz, CD_3_OD) δ_c_ 195.5, 164.5, 164.1, 139.8, 111.9, 110.4, 102.4, 56.5 (S9 and S10 Figs).

### Ethyl 4-methoxybenzofuran-2-carboxylate (6)

A mixture of **5** (100 g, 660 mmol), ethyl bromoacetate (210 g, 1.27 mol) and K_2_CO_3_ (270 g, 1.95 mol) in DMF (600 ml) was heated to 140 °C. After 4 h, TLC showed that the reaction was complete. The mixture was cooled to room temperature, and water (2 L) was added. The mixture was extracted with EtOAc (300 ml) twice and concentrated in vacuo. The residue was purified by column chromatography on silica (100–200 mesh) with petroleum ether to provide compound **6** (117 g, 55% yield); yellow solid^35^; mp 135–140 °C. ^1^H-NMR(600 MHz, CD_3_OD) δ_H_ 7.54(1 H, d, J = 0.9 Hz, Ar-H), 7.38 (1H, t, J = 8.2 Hz, Ar-H), 7.12 (1H, d, J = 8.4 Hz, Ar-H), 6.75 (1H, d, J = 8.0 Hz, Ar-H), 4.39 (2H, J = 7.1 Hz), 3.93 (3H, s, OCH_3_), 1.39 (3H, t, J = 7.1 Hz); ^13^C-NMR (150 MHz, CD_3_OD) δ_c_ 160.8, 158.1, 156.0, 145.5, 130.0, 118.8, 112.2, 105.5, 104.7, 62.4, 56.2, 14.5 (S11 and S12 Figs).

### 4-Methoxybenzofuran-2-carboxy (7)

**A** solution of **6** (60 g, 272 mmol) in MeOH (500 ml) and NaOH (2 mol/L, 300 ml) was stirred at 100 °C for 30 min. The mixture was cooled to room temperature, and aqueous 1 N HCl (500 ml) was added to adjust the pH to 1. Extraction was performed with EtOAc (300 ml) twice, and the extracts were concentrated in vacuo to give product **7** (50 g, 85% yield); white powder^36^; mp 205–210 °C. ^1^H-NMR(600 MHz, CD_3_OD) δ_H_ 7.58(1H, d, J = 0.8 Hz, Ar-H), 7.41 (1H, t, J = 8.3 Hz, Ar-H), 7.16 (1H, d, J = 8.4 Hz, Ar-H), 6.79 (1H, d, J = 8.0 Hz, Ar-H), 3.96 (3H, s, OCH_3_); ^13^C-NMR(150 MHz, CD_3_OD) δ_c_ 162.3, 158.2, 156.1, 146.0, 129.9, 119.0, 112.2, 105.6, 104.7, 56.2 (S13 and S14 Figs).

### 2,3-Dihydro-2-carboxy-4-methoxybenzofuran (8)

Compound **7** (30 g, 156 mmol) was diluted with MeOH (1 L) and stirred below 30 °C, Magnesium was added until the reaction finished. The reaction residue was added to aqueous 1 N HCl (500 ml) to adjust pH to 1, extracted with EtOAc (500 ml) thrice and concentrated in vacuo to give product **8** (28 g, 98% yield); yellow oil^37^. ^1^H-NMR (400 MHz, CD_3_OD) δ_H_ 7.07 (1H, t, J = 8.0 Hz, Ar-H), 6.45 (2H, m, Ar-H), 5.17 (1H, m), 3.78(3H, s, OCH_3_), 3.30(1H, m), 3.20 (1H, m); ^13^C-NMR (100 MHz, CD_3_OD) δ_c_ 175.2, 161.7, 157.9, 130.5, 113.0, 104.6, 103.6, 80.3, 55.8, 32.4 (S15 and S16 Figs).

### (*S)*-(-)-Methylbenzylammonium(*S*)-2,3-dihydro-4-methoxybenzofuran-2-carboxylate (9a)

To a solution of **8** (10 g, 51.8 mmol) in Me_2_CO (20 ml) was added 6.8 ml (51.8 mmol) of (*S*)-(-)-α-MBA was added, and the solution was stirred for 10 min. The white precipitate formed was filtered and washed with Me_2_CO to give the homogeneous salt (*S*)-(-)-**9a** (4.9 g, 98% yield); white solid; mp 155–160 °C; [R]_D_^20^ −4.83° (*c* 0.1, MeOH)^38^ (S35 Fig).

### (*R*)-(+)-Methylbenzylammonium(*R*)-2,3-dihydro-4-methoxybenzofuran-2-carboxylate(9b)

Using the above procedure with 1 eq of (*R*)-(+)-α-MBA, gave 4.9 g of the salt (*R*)-(+)-**9b** as a white amorphous solid; 98% yield; mp 155–160 °C; [R]_D_^20^ + 4.83° (*c* 0.1, MeOH) (S36 Fig).

### (*S*)-(-)-2,3-Dihydro-2-carboxy-4-methoxybenzofuran (8a)

To a vigorously stirred mixture of the (*S*)-(-)-9a (10 g), water (60 mL) and EtOAc (60 ml) at 0 °C, aqueous 5 N HCl (20 ml) was added dropwise over a period of 20 min. The separated organic layer was evaporated under vacuum to afford (*S*)-(-)-**8a** (6 g, 98% yield); white solid; mp 125–130 °C; [R]_D_^20^ −6.33° (*c* 0.1, MeOH).

### (*R*)-(+)-2,3-Dihydro-2-carboxy-4-methoxybenzofuran (8b)

Using the above procedure, gave (*R*)-(+)-**8b** (6 g, 98% yield); white solid; mp 125–130 °C; mp 125–130 °C; [R]_D_^20^ + 6.33° (*c* 0.1, MeOH).

### (*S*)-(-)-2,3-Dihydro-2-carboxamide-N-methoxy-N-methyl-4-methoxybenzofuran (10)

To a solution of **8a** (40 g, 206 mmol) in anhydrous Cl_2_CH_2_ (500 ml), under nitrogen, 1,1′-carbonyldiimidazole (37 g, 226 mmol) was added. After stirring for 1 h, N,O-dimethylhydroxylamine hydrochloride (23 g, 226 mmol) was added portion-wise and further stirred at room temperature overnight. Then, the mixture was washed with aqueous 1 N HCl (500 ml) and 10% K_2_CO_3_ (500 ml) twice, respectively. The organic phase was separated, dried (Na_2_SO_4_) and evaporated under vacuum. The residue was purified by column chromatography on silica (100–200 mesh; petroleum ether then 20% EtOAc/petroleum ether) to afford compound **10** (44 g, 89% yield); grey solid^39^; mp 115–120 °C; [R]_D_^20^ −8.03° (*c* 0.1, MeOH). ^1^H-NMR (600 MHz, CD_3_OD) δ_H_ 7.10 (1H, t, J = 8.1 Hz, Ar-H), 6.49 (1H, d, J = 8.3 Hz,Ar-H), 6.45 (1H, d, J = 8.0 Hz, Ar-H), 5.60(1H, t, J = 7.9 Hz), 3.82 (6H, s, OCH_3_), 3.47(1H, m), 3.25 (3H, s, NCH_3_), 3.16 (1H, m); ^13^C-NMR (150 MHz, CD_3_OD) δ_c_ 173.3, 161.9, 157.9, 130.4, 113.4, 104.5, 103.6, 81.1, 79.6, 62.1, 55.8, 32.6 (S17 and S18 Figs).

### (*S*)-(-)-2,3-Dihydro-2-carboxamide-N-methoxy-N-methyl-4-methoxybenzofuran-7-iodo (11)

To a solution of **10** (32 g, 134 mmol) and silver trifluoromethanesulfonate (34.5 g, 134 mmol) in CHCl_3_ (500 ml), iodine (34 g, 134 mmol) was added portion-wise with vigorous stirring at room temperature over a period of 1 min. Whenthe iodine disappeared, the mixture was passed through Celite, and the filtrate was washed with saturated aqueous Na_2_S_2_O_3_, dried (Na_2_SO_4_) and concentrated under vacuum. The resulting crude product was purified by silica (100–200 mesh) column chromatography (eluting with petroleum ether) to obtain pure compound **11** (27 g, 55% yield); white solid; mp 113–118 °C; [R]_D_^20^ −9.62° (*c* 0.1, MeOH). ^1^H-NMR (600 MHz, CD_3_OD) δ_H_ 7.41 (1H, d, J = 8.6 Hz, Ar-H), 6.39 (1 H, d, J = 8.6 Hz, Ar-H), 5.68 (1H, t, J = 7.6 Hz), 3.83(3H, s, OCH_3_),3.82(3H, s, OCH_3_), 3.39(1H, m), 3.33 (1H, m), 3.32 (3H, s, NCH_3_);^13^C-NMR(150 MHz, CD_3_OD) δ_c_ 158.3, 138.7, 114.2, 107.3, 79.4, 63.4, 56.1, 32.6 (S19, S20 and S21 Figs).

### (*S*)-(-)-2,3-Dihydro-2-carboxamide-N-methoxy-N-methyl-4-methoxybenzofuran-5-bromo-7-iodo (12)

To a solution of **11** (37 g, 101 mmol) in THF (100 ml), N-bromosuccinmide (20 g, 112 mmol) was added portion-wise with vigorous stirring at room temperature over a period of 10 min. The whole was directly concentrated under vacuum. The residue was purified by the above procedure to provide compound **12** (32 g, 70% yield); white amorphous solid^40^; mp 80–85 °C; [R]_D_^20^ −9.42° (*c* 0.1, MeOH). ^1^H-NMR (600 MHz, CD_3_OD) δ_H_ 7.63 (1H, s, Ar-H), 5.74(1H, t, J = 7.2 Hz), 3.90 (6H, s, OCH_3_), 3.76 (1H, m), 3.36 (1H, m), 3.32 (3H, s, NCH_3_); ^13^C-NMR (150 MHz, CD_3_OD) δ_c_ 171.5, 155.0, 140.8, 135.5, 119.0, 108.2, 97.4, 80.4, 66.7, 62.6, 60.3, 32.6 (S22, S23 and S24 Figs).

### (*S*)-(-)-2,3-Dihydro-2-ethanone-4-methoxybenzofuran-5-bromo (13)

To stirring anhydrous THF (60 ml) at −10 °C, methylmagnesium bromide (27 g, 226 mmol) was added dropwise over a period of 10 min. The mixture was added portion-wise to **12** (10 g, 22.6 mmol) below 0 °C under nitrogen. TLC showed that the reaction was completed, and the reaction was quenched with saturated aqurous NH_4_Cl (50 ml). The organic phase was separated and evaporated in vacuo. The oily residue was purified on a silica gel column (100–200 mesh) with petroleum ether to provide compound **13** (4.5 g, 73% yield); yellow oil^41^; [R]_D_^20^ −5.31° (*c* 0.1, MeOH). ^1^H-NMR (600 MHz, CD_3_OD) δ_H_ 7.30 (1H, d, J = 8.4 Hz, Ar-H), 6.52 (1H, d, J = 8.4 Hz, Ar-H), 5.20 (1H, m), 3.88 (3H, s, OCH_3_), 3.62 (1H, m), 3.44 (1H, m), 2.27 (3H, s, CH_3_);^ 13^C-NMR (150 MHz, CD_3_OD)δ_c_ 208.7, 162.0, 154.7, 135.0, 118.8, 108.8, 107.1, 87.9, 60.1, 31.8, 26.1 (S25 and S26 Figs).

### (*S*)-(-)-2,3-Dihydro-2-isopropenyl-4-methoxybenzofuran-5-bromo (14)

Potassium tert-butoxide (34 g, 303 mmol) was added to the suspension of methyl triphenylphosphonium iodide (120 g, 297 mmol) in anhydrous diethyl ether (500 ml) in ice-cooled conditions. After being stirred for 1 h, a solution of **13** (10 g, 37 mmol) in diethyl ether (50 ml) was added dropwise. The mixture was stirred at 40 °C overnight, and water (50 ml) was slowly added to quench the reaction. After the evaporation of volatiles, the resulting residue was purified by column chromatography on silica (100–200 mesh) with petroleum ether to afford compound **14** (6.6 g, 65% yield); yellow oil^42^; [R]_D_^20^ −5.42° (*c* 0.1, MeOH). ^1^H-NMR (600 MHz, CD_3_OD) δ_H_ 6.60 (1H, d, J = 8.4 Hz, Ar-H), 6.30 (1H, d, J = 8.4 Hz, Ar-H), 5.12 (1H, t, J = 8.6Hz), 5.07 (1H, s), 4.89 (1H, s), 3.86(3H, s, OCH_3_), 3.44(1H, m), 3.05 (1H, m), 1.77 (3H, s, CH_3_); ^13^C-NMR (150 MHz, CD_3_OD) δ_c_ 155.2, 145.9, 145.7, 143.9, 118.8, 116.1, 111.9, 104.2, 87.1, 60.0, 34.4, 17.3 (S27 and S28 Figs).

### (*S*)-(-)-2,3-Dihydro-2-isopropenyl-4-methoxybenzofuran-5-boronic acid pinacol ester (15)

To a stirring solution of **14** (24 g, 89 mmol) in dimethyl sulphoxide (500 ml), KOAc (44 g, 449 mmol), bis(pinacolato)diboron (35 g, 138 mmol) and Pb(dppf)_2_Cl_2_ (8 g, 9 mmol) were added. The reaction vessel was vacuumed and backfilled with nitrogen five times. The whole was stirred at 105 °C overnight. The mixture was passed through Celite, and the filtrate was added to water (1 L), extracted with EtOAc (500 ml) thrice and concentrated in vacuo. The residue was purified by flash chromatography with petroleum ether to provide compound **15** (12 g, 43% yield); yellow amorphous solid^43^; mp 103–108 °C; [R]_D_^20^ −10.02° (*c* 0.1, MeOH). ^1^H-NMR (600 MHz, CDCl_3_) δ_H_ 7.58 (1H, d, J = 8.0 Hz, Ar-H), 6.59 (1H, d, J = 8.0 Hz, Ar-H), 5.21 (1H, t, J = 8.4 Hz), 5.09 (1H, s), 4.92 (1H, s), 3.85 (3H, s, OCH_3_), 3.40 (1 H, m), 3.05 (1H, m), 1.76 (3H, s, CH_3_), 1.32 (12H, s, CH_3_); ^13^C-NMR (150 MHz, CDCl_3_) δ_c_ 164.3, 162.7, 143.6, 137.8, 117.4, 112.1, 104.8, 86.3, 83.2, 60.9, 32.4, 24.8, 24.8, 17.0 (S29 and S30 Figs).

### 7-Hydroxy-2′-methoxy-2′′ S-isopropenyl dihydrofuran [6′′, 7′′ : 3′, 4′]-isoflavone (16)

To a stirring solution of **3** (5 g, 16 mmol) and **15** (2.5 g, 8 mmol) in 1,4-dioxane (150 ml), aqueous 1 N Na_2_CO_3_ (50 ml) and Pb(dppf)_2_Cl_2_ (1.3 g, 1.5 mmol) were added at room temperature. The reaction vessel was vacuumed and backfilled with nitrogen five times. The mixture was further stirred at 50 °C overnight under nitrogen. The reaction mixture was filtered through a pad of Celite, diluted with water (200 ml) and extracted with EtOAc. The combined extracts were dried over Na_2_SO4 and concentrated. Purification by flash chromatography (petroleum ether then 20% EtOAc/petroleum ether) afforded compound **16** (2.1 g, 70% yield) as a yellow needles. ^1^H-NMR (600 MHz, pyridine-*d*_*5*_) δ_H_ 8.70 (1H, s), 8.43 (1H, d, J = 8.7 Hz, Ar-H), 7.26 (1H, d, J = 8.0 Hz, Ar-H), 7.19 (1H, dd, J = 8.1, 2.2 Hz, Ar-H), 7.08 (1 H, d, J = 2.2 Hz, Ar-H), 6.74 (1 H, d, J = 8.0 Hz, Ar-H), 5.21(1 H, t, J = 8.8 Hz), 5.17 (1H, s), 4.91 (1H, s), 3.79 (3H, s, OCH_3_), 3.40 (1H, m), 3.12 (1H, m), 1.71 (3H, s, CH_3_); ^13^C-NMR (150 MHz, pyridine-*d*_*5*_) δ_c_ 175.5, 163.8, 162.3, 158.5, 153.3, 149.9, 144.2, 131.9, 127.9, 122.9, 117.6, 117.3, 116.5, 115.5, 111.8, 103.9, 102.9, 86.1, 58.9, 33.6, 17.0 (S31 and S32 Figs).

### 7, 2′- Dihydroxy-2′′ S-isopropenyl dihydrofuran [6′′, 7′′ : 3′, 4′]-isoflavone (17)

To a solution of **16** (1 g, 2.8 mmol), ethanethiol sodiumsalt (1:1) (0.8 g, 8.3 mmol) in DMF (20 ml) was added. The mixture was further stirred at 100 °C overnight, diluted with water (50 ml) and extracted with EtOAc. The combined organic extracts were washed with brine, dried (Na_2_SO_4_), filtered and concentrated. Purification of the residue by flash chromatography (petroleum ether then 20% EtOAc/petroleum ether) provided compound **17** (2.1 g, 70% yield); yellow needles^44^; mp 155–160 °C; [R]_D_^20^ −8.74° (*c* 0.1, MeOH). ^1^H-NMR (600 MHz, CD_3_OD) δ_H_ 8.13 (1H, s), 8.07 (1H, d, J = 8.8 Hz, Ar-H), 6.97 (1H, dd, J = 9.0, 1.8 Hz, Ar-H), 6.96 (1H, d, J = 7.8 Hz, Ar-H), 6.88 (1H, d, J = 2.0 Hz, Ar-H), 6.37 (1H, d, J = 8.2 Hz, Ar-H), 5.23 (1H, t, J = 8.7 Hz), 5.08 (1H, s), 4.91 (1H, s), 3.35 (1H, m), 2.98 (1H, m), 1.78 (3H, s, CH_3_); ^13^C-NMR (150 MHz, CD_3_OD) δ_c_ 179.4, 164.8, 163.2, 159.7, 156.5, 153.8, 145.9, 131.9, 130.2, 129.3, 128.5, 124.5, 117.6, 116.7, 115.1, 111.9, 102.4, 87.7, 33.3, 17.2 (S33 and S34 Figs).

### Animals and diets

Six-week-old female C57/BL6 mice were purchased from the SLAC laboratory and housed in a temperature-controlled and pathogen-free room under a 12:12-h light-dark cycle with free access to food and water until the start of the experiments. We fed thirty seven-week-old C57/BL6 mice with HFD to induce obesity for three months and ten mice with LFD as a chow group. The obese mice were randomly fed with CC-1 (50 mg/kg) or CC-2 (100 mg/kg) milled in HFD or regular HFD for 20 days (n = 10). At the end of the study period, all mice were fasted for 12 h and then scarified. Blood and tissue samples were collected and either fixed in 4% paraformaldehyde or snap frozen and stored at −80 °C.

All animal experiments and protocols met the requirements of the Animal Ethics Committee and approved by Shanghai University of Traditional Chinese Medicine in accordance with the Guide for the Care and Use of Laboratory Animals (Approved Number: SZY201604003).

### Metabolic Analyses

Body weight and food consumption were recorded every two days, and the body composition was measured by nuclear magnetic resonance (NMR) spectroscopy. For the intraperitoneal glucose tolerance test (ipGTT) and intraperitoneal insulin tolerance test (ipITT), all mice were received with 1 g/kg glucose and 0.75 U/kg insulin (Sigma, St. Louis, MO). The total cholesterol, TG, insulin, leptin, adiponectin, ALT and AST of serum and hepatic lipids were detected as described previously^20^.

### Inflammatory markers assay in serum

Serum TNF-α, IL-1β and IL-6 were measured using enzyme-linked immunosorbent assay according to the manufacturer’s instructions (ebioscience, USA). All determinations were performed in duplicates.

### Histochemistry

The fresh liver samples and white adipose tissue were fixed in 4% paraformaldehyde, embedded in paraffin, sectioned into 5 μm and stained with haematoxylin and eosin (H&E). For studying lipid accumulation in the liver, liver samples were frozen, embedded in O.C.T. compound, sliced into 8 μm, and then stained with Oil Red O. All protocols are in accordance with a standard procedure.

### TR-FRET assay

A LanthaScreen time-resolved fluorescence resonance energy transfer (TR-FRET) competitive binding assay was performed according to the manufacturer’s (Invitrogen, Germany) protocol. In brief, the assay was conducted in black noncoated low-volume round-bottomed 384-well Coring plates. The interaction between the ligand-binding domain (LBD) of human nuclear receptors tagged with glutathione S-transferase (GST), the terbium labelled anti-GST antibody and the fluorescent small molecule was measured by a PerkinElmer Envision plate reader (PerkinElmer, Waltham, MA). For the TR-FRET ratios, the emission signal at 520 nm was divided by the emission signal at 495 nm.

### Computational molecular docking

The crystal structure of PPARγ (PDB code 2I4J) was retrieved from the Research Collaborator for Structural Bioinformatics (RCSB) Protein Bank. Docking was conducted using MOE2012.10.

### RNA isolation and quantitative real-time PCR

The total RNA from liver and adipose tissue was isolated using the Trizol reagent (TaKaRa, Japan). Oligo-dT-primed cDNA synthesis was carried out with 3 μg of total RNA using a cDNA synthesis kit (Thermo, America). Quantitative real-time assays for mRNA measurements were performed using SYBR green in an ABI StepOnePlus Real-Time PCR system (Applied Biosystems, Foster City, CA) with primer sequences as listed in Table 1. All final expressions of target genes were calculated relative to the housekeeping gene β-actin.

### Statistical Analysis

All data are presented as a mean ± SEM. Individual pairwise comparisons were analysed by paired or unpaired two tailed t-tests. Two-way analysis of variance (ANOVA) was used for multiple comparisons. Differences with P values < 0.05 were considered statistically significant.

## Additional Information

**How to cite this article:** Sun, Q.-H. *et al*. Synthesis and anti-obesity effects *in vivo* of Crotadihydrofuran C as a novel PPARγ antagonist from *Crotalaria albida. Sci. Rep.* **7**, 46735; doi: 10.1038/srep46735 (2017).

**Publisher's note:** Springer Nature remains neutral with regard to jurisdictional claims in published maps and institutional affiliations.

## Supplementary Material

Supporting Information

## Figures and Tables

**Table 1:** Sequences of the primers used in real time PCR

Gene	Forward primer	Reverse primer
β-Actin	TGTCCACCTTCCAGCAGATGT	AGCTCAGTAACAGTCCGCCTAGA
FAS	CTGAGATCCCAGCACTTCTTGA	GCCTCCGAAGCCAAATGAG
LPL	ATCGGAGAACTGCTCATGATGA	CGGATCCTCTCGATGACGAA
aP2	CATGGCCAAGCCCAACAT	CGCCCAGTTTGAAGGAAATC
ACC	GAATCTCCTGGTGACAATGCTTATT	GGTCTTGCTGAGTTGGGTTAGCT
CD36	GCTTGCAACTGTCAGCACAT	GCCTTGCTGTAGCCAAGAAC
PPARγ	TGCTGTATTTGAATCCGACGTT	GCTCTTTAGAAACTCCCTTGTCATG
SREBP-1c	GGCTATTCCGTGAACATCTCCTA	ATCCAAGGGCAGTTCTTGTG
SCD-1	TCACCTTGAGAGAAGAATTAGCA	TTCCCATTCCCTTCACTCTGA
IL-1β	TCGTGCTGTCGGACCCATAT	GGTTCTCCTTGTACAAAGCTCATG
IL-4	GCAGAGACTCTTTCGGGCTTT	CATTCATGGTGCAGCTTATCGA
IL-6	AACCACGGGCTTCCCTACTT	TCTGTTGGGAGTGGTATCCTCTGT
IL-10	GCCAAGCCTTATCGGAAATG	CTTGATTTCTGGGCCATGCT
TNF-α	ATGGATCTCAAAGACAACCAACTAG	ACGGCAGAGAGGAGGTTGACTT
CD68	TCACCTTGACCTGCTCTCTCTAA	GCTGGTAGGTTGATTGTCGTCTG
α−ΣΜΑ	GTCCCAGACATCAGGGAGTAA	TCGGATACTTCAGCGTCAGGA
Col1a	ACGTCCTGGTGAAGTTGGTC	CAGGGAAGCCTCTTTCTCCT
Col3a	GTTCTAGAGGATGGCTGTACTAAA	TTGCCTTGCGTGTTTGATATTC
TIMP1	CATGGAAAGCCTCTGTGGATATG	AAGCTGCAGGCACTGATGTG
Mmp9	CGAACTTCGACACTGACAAGAAGT	GCACGCTGGAATGATCTAAGC
