# Supplementary material for: Synthesis and anti-obesity effects in vivo of Crotadihydrofuran C as a novel PPARγ antagonist from Crotalaria albida
Source: Sci Rep. 2017 Apr 24;7:46735. doi: 10.1038/srep46735 (PMC5402262; doi:10.1038/srep46735)
Supplement: Supporting Information [file srep46735-s1.doc]

Synthesis and anti-obesity effects in vivo of Crotadihydrofuran C as a novel PPARγ antagonist from *Crotalaria albida*

Qin-Hu Sun, ^1, 2^ Yu Zhang, ^3^ Gui-Xin Chou ^*, 1, 2^

^1^ The MOE Key Laboratory for Standardization of Chinese Medicines and SATCM Key Laboratory for New Resources and Quality Evaluation of Chinese Medicines, Institute of Chinese Materia Medica of Shanghai University of Traditional Chinese Medicine, Cai Lun Road 1200, Zhangjiang, Shanghai, 201210, People’s Republic of China

^2^ Shanghai R&D Center for Standardization of Chinese Medicines, Shanghai 201203, People’s Republic of China

^3^ School of Life Science and Technology, ShanghaiTech University, Shanghai 201210, China

* Corresponding authors: Guixin Chou

Fax: +86-21-50271708; Tel: +86-21-50271706

E-mail addresses: chouguixinzyb@126.com

Supporting Information:

S1 Fig. ^1^H-NMR spectrum (400 MHz, CD_3_OD) of 1.

S2 Fig. ^13^C-NMR spectrum (100 MHz, CD_3_OD) of 1

S3 Fig. ^1^H-NMR spectrum (400 MHz, CDCl_3_) of 2.

S4 Fig. ^13^C-NMR spectrum (100 MHz, CDCl_3_) of 2.

S5 Fig. ^1^H-NMR spectrum (600 MHz, CD_3_OD) of 3.

S6 Fig. ^13^C-NMR spectrum (150 MHz, CD_3_OD) of 3.

S7 Fig. ^1^H-NMR spectrum (600 MHz, CD_3_OD) of 4.

S8 Fig. ^13^C-NMR spectrum (150 MHz, CD_3_OD) of 4.

S9 Fig. ^1^H-NMR spectrum (600 MHz, CD_3_OD) of 5.

S10 Fig. ^13^C-NMR spectrum (150 MHz, CD_3_OD) of 5.

S11 Fig. ^1^H-NMR spectrum (600 MHz, CD_3_OD) of 6.

S12 Fig. ^13^C-NMR spectrum (150 MHz, CD_3_OD) of 6.

S13 Fig. ^1^H-NMR spectrum (600 MHz, CD_3_OD) of 7.

S14 Fig. ^13^C-NMR spectrum (150 MHz, CD_3_OD) of 7.

S15 Fig. ^1^H-NMR spectrum (400 MHz, CD_3_OD) of 8.

S16 Fig. ^13^C-NMR spectrum (100 MHz, CD_3_OD) of 8.

S17 Fig. ^1^H-NMR spectrum (600 MHz, CD_3_OD) of 10.

S18 Fig. ^13^C-NMR spectrum (150 MHz, CD_3_OD) of 10.

S19 Fig. ^1^H-NMR spectrum (600 MHz, CD_3_OD) of 11.

S20 Fig. ^13^C-NMR spectrum (150 MHz, CD_3_OD) of 11.

S21 Fig. NOESY spectrum of 11.

S22 Fig. ^1^H-NMR spectrum (600 MHz, CD_3_OD) of 12.

S23 Fig. ^13^C-NMR spectrum (150 MHz, CD_3_OD) of 12.

S24 Fig. NOESY spectrum of 12.

S25 Fig. ^1^H-NMR spectrum (600 MHz, CD_3_OD) of 13.

S26 Fig. ^13^C-NMR spectrum (150 MHz, CD_3_OD) of 13.

S27 Fig. ^1^H-NMR spectrum (600 MHz, CD_3_OD) of 14.

S28 Fig. ^13^C-NMR spectrum (150 MHz, CD_3_OD) of 14.

S29 Fig. ^1^H-NMR spectrum (600 MHz, CDCl_3_) of 15.

S30 Fig. ^13^C-NMR spectrum (150 MHz, CDCl_3_) of 15.

S31 Fig. ^1^H-NMR spectrum (600 MHz, Pyridine-*d_5_*) of 16.

S32 Fig. ^13^C-NMR spectrum (150 MHz, Pyridine-*d_5_*) of 16.

S33 Fig. ^1^H-NMR spectrum (600 MHz, CD_3_OD) of 17.

S34 Fig. ^13^C-NMR spectrum (150 MHz, CD_3_OD) of 17.

S35 Fig. Ord result of 9a

S36 Fig. Ord result of 9b


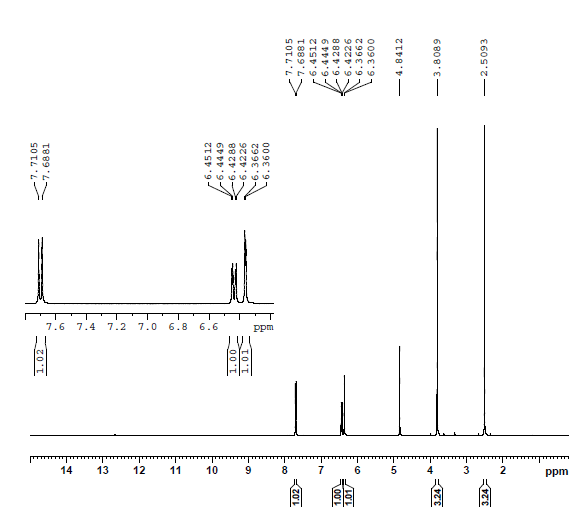


**S1 Fig. ^1^H-NMR spectrum (400 MHz, CD_3_OD) of 1.**


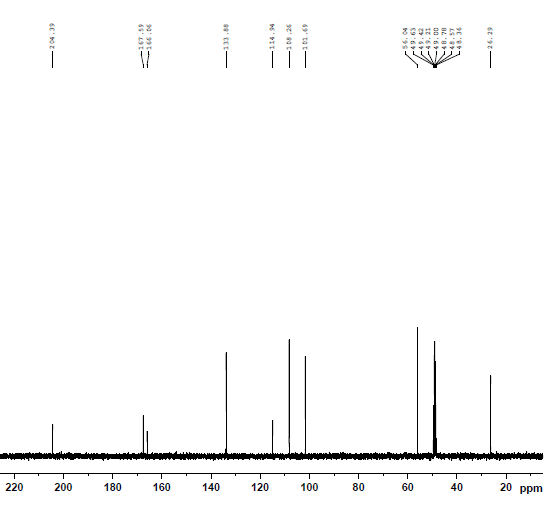


**S2 Fig. ^13^C-NMR spectrum (100 MHz, CD_3_OD) of 1**


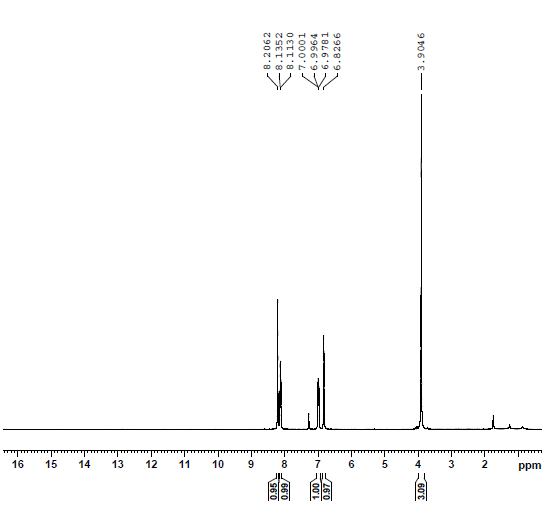


**S3 Fig. ^1^H-NMR spectrum (400 MHz, CDCl_3_) of 2.**


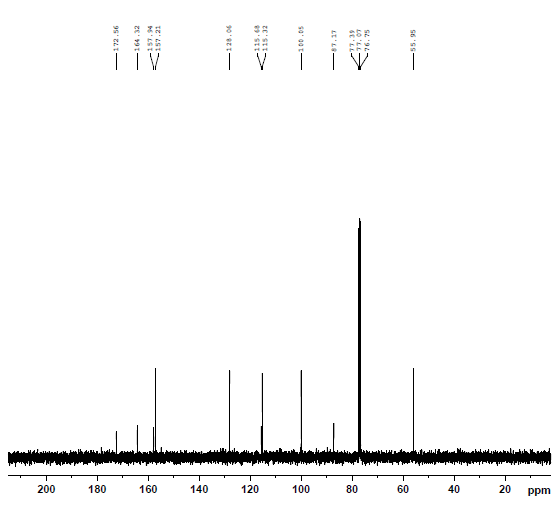


**S4 Fig. ^13^C-NMR spectrum (100 MHz, CDCl_3_) of 2.**


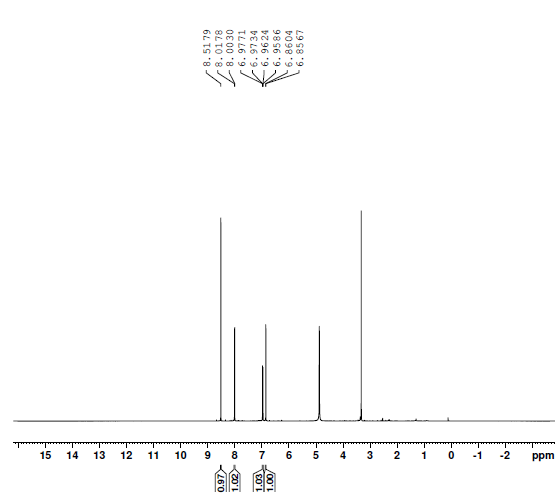


**S5 Fig. ^1^H-NMR spectrum (600 MHz, CD_3_OD) of 3.**


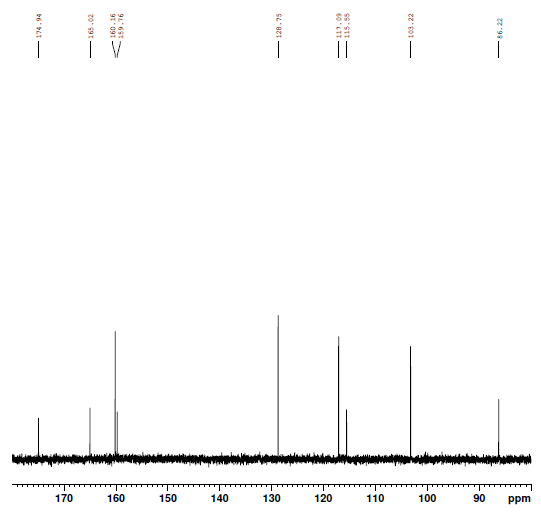


**S6 Fig. ^13^C-NMR spectrum (150 MHz, CD_3_OD) of 3.**


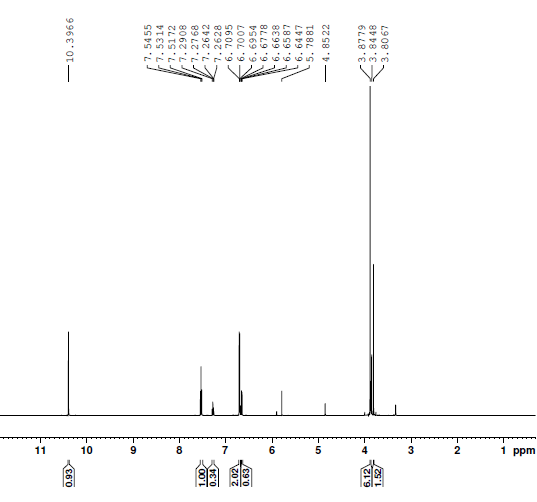


**S7 Fig. ^1^H-NMR spectrum (600 MHz, CD_3_OD) of 4.**


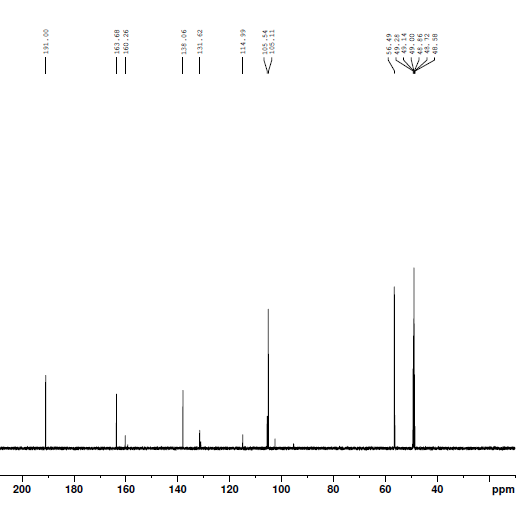


**S8 Fig. ^13^C-NMR spectrum (150 MHz, CD_3_OD) of 4.**


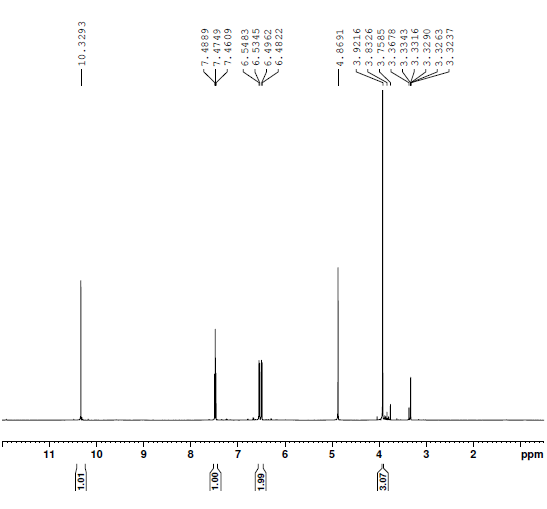


**S9 Fig. ^1^H-NMR spectrum (600 MHz, CD_3_OD) of 5.**


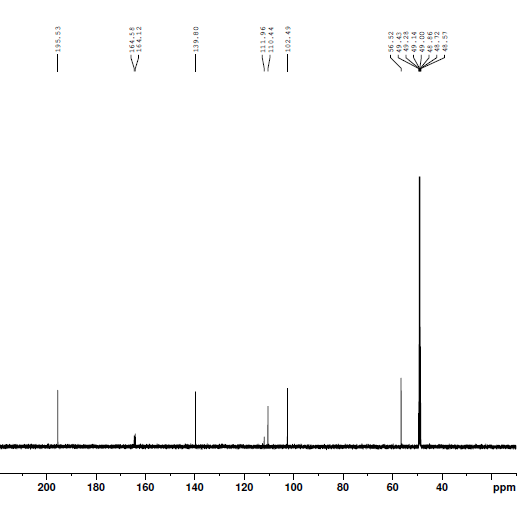


**S10 Fig. ^13^C-NMR spectrum (150 MHz, CD_3_OD) of 5.**


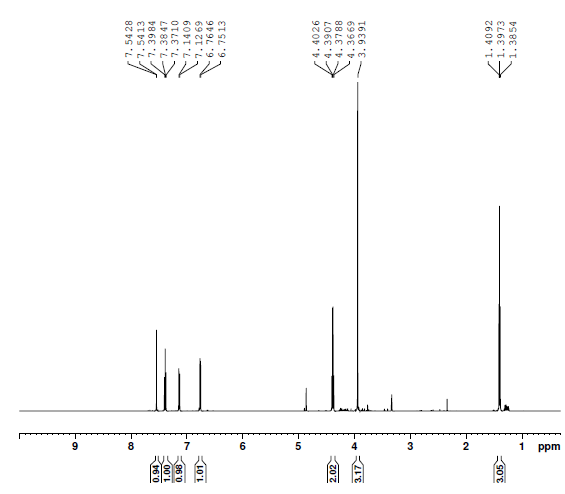


**S11 Fig. ^1^H-NMR spectrum (600 MHz, CD_3_OD) of 6.**


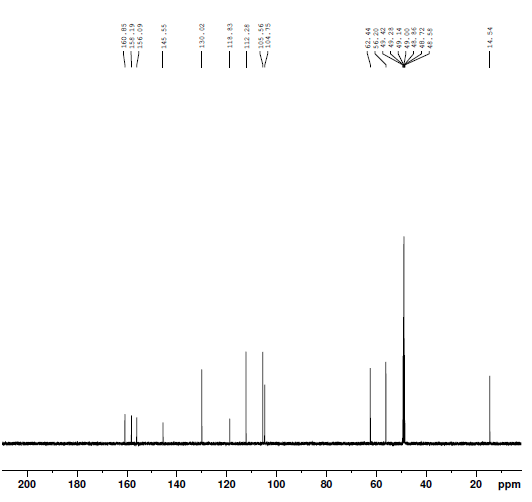


**S12 Fig. ^13^C-NMR spectrum (150 MHz, CD_3_OD) of 6.**


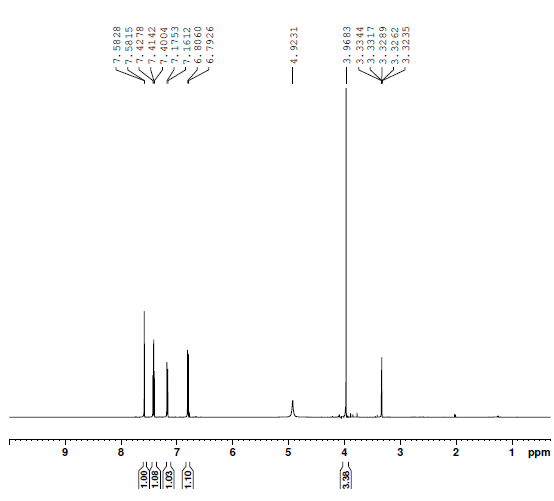


**S13 Fig. ^1^H-NMR spectrum (600 MHz, CD_3_OD) of 7**


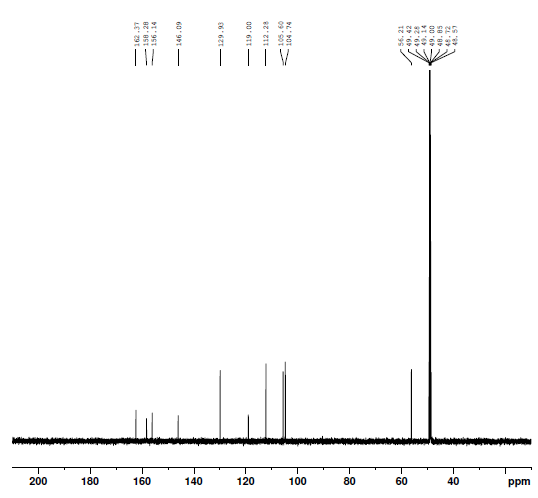


**S14 Fig. ^13^C-NMR spectrum (150 MHz, CD_3_OD) of 7**


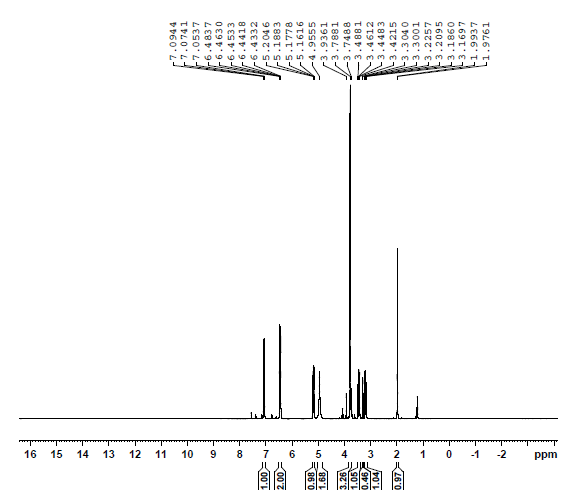


**S15 Fig. ^1^H-NMR spectrum (400 MHz, CD_3_OD) of 8**


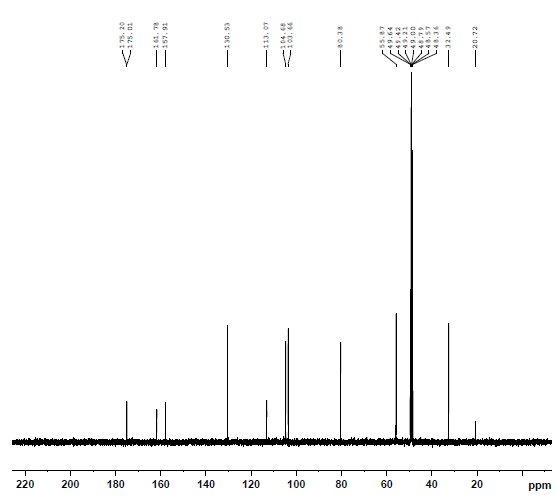


**S16 Fig. ^13^C-NMR spectrum (100 MHz, CD_3_OD) of 8.**


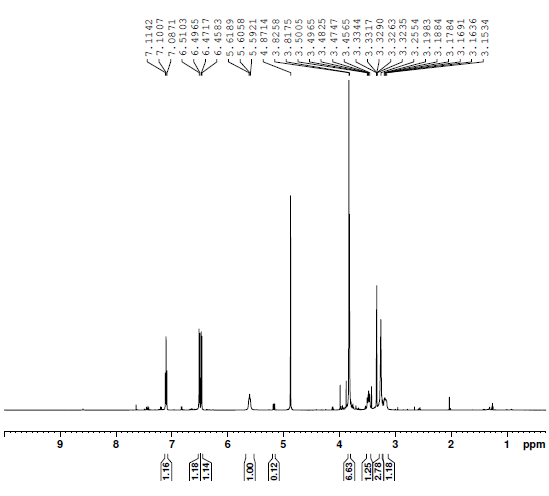


**S17 Fig. ^1^H-NMR spectrum (600 MHz, CD_3_OD) of 10**


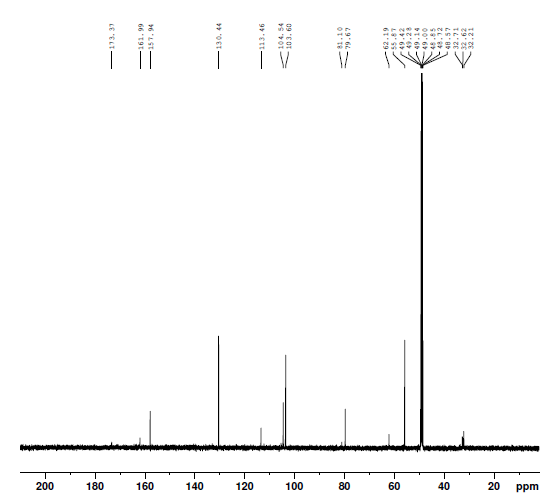


**S18 Fig. ^13^C-NMR spectrum (150 MHz, CD_3_OD) of 10**


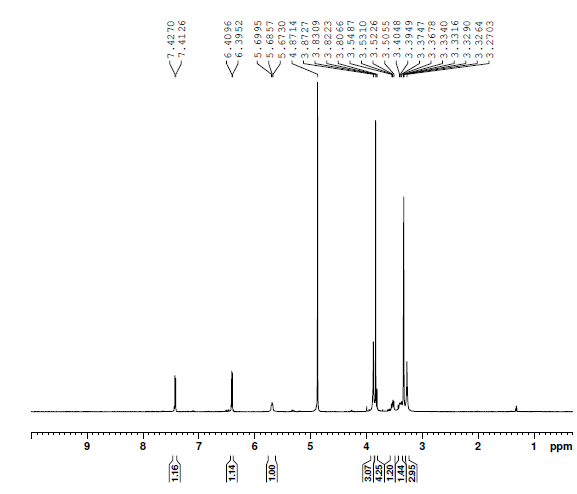


**S19 Fig. ^1^H-NMR spectrum (600 MHz, CD_3_OD) of 11**


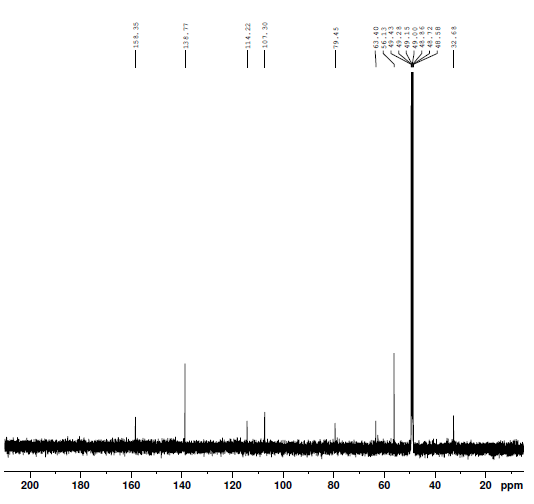


**S20 Fig. ^13^C-NMR spectrum (150 MHz, CD_3_OD) of 11**


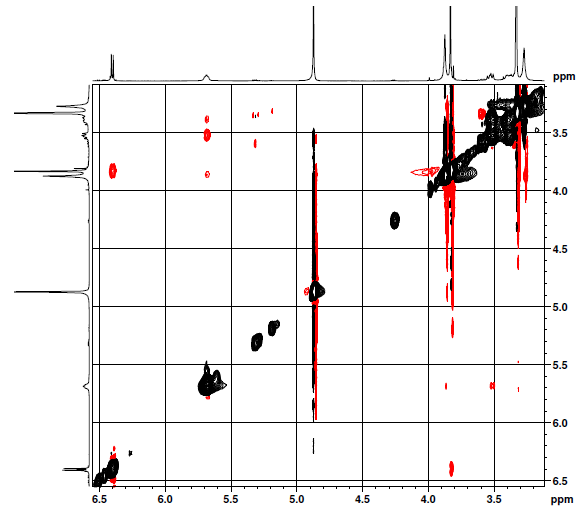


**S21 Fig. NOESY spectrum of 11.**


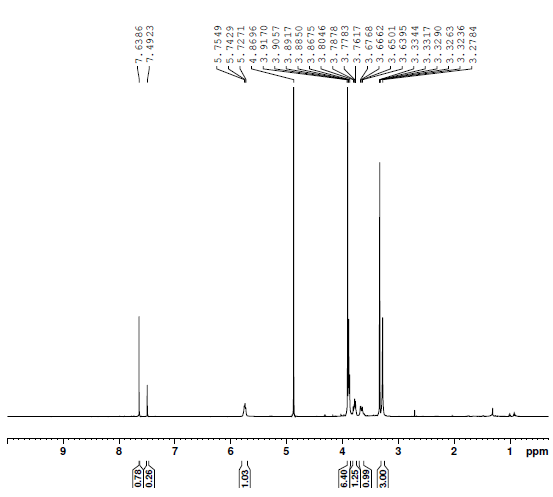


**S22 Fig. ^1^H-NMR spectrum (600 MHz, CD_3_OD) of 12**


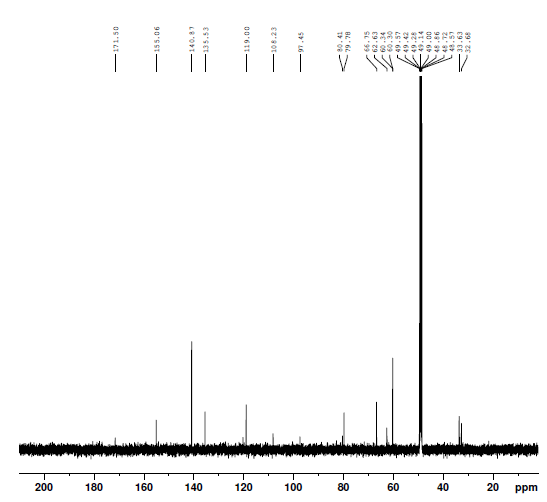


**S23 Fig. ^13^C-NMR spectrum (150 MHz, CD_3_OD) of 12**


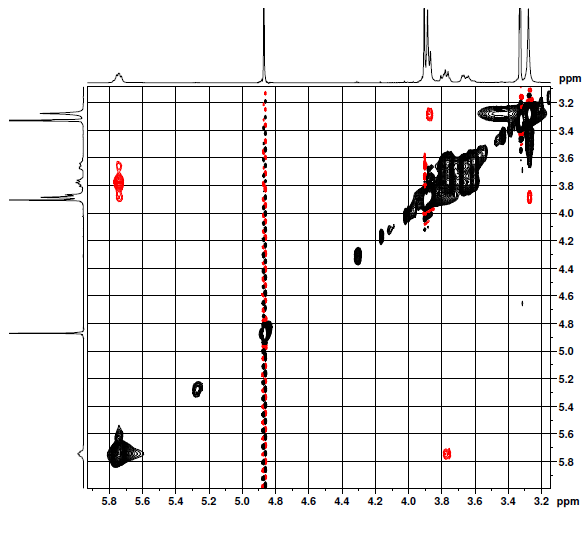


**S24 Fig. NOESY spectrum of 12**


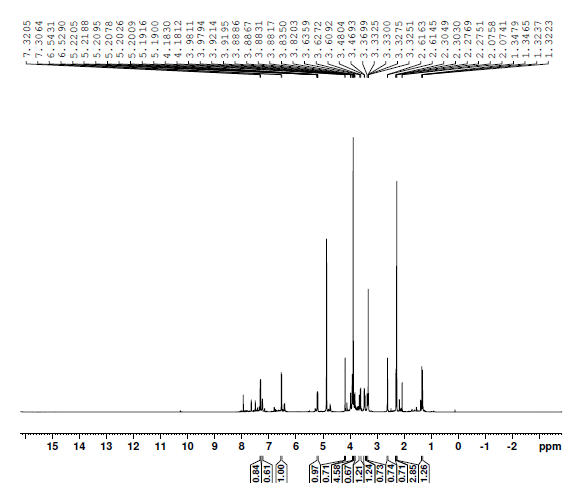


**S25 Fig. ^1^H-NMR spectrum (600 MHz, CD_3_OD) of 13**


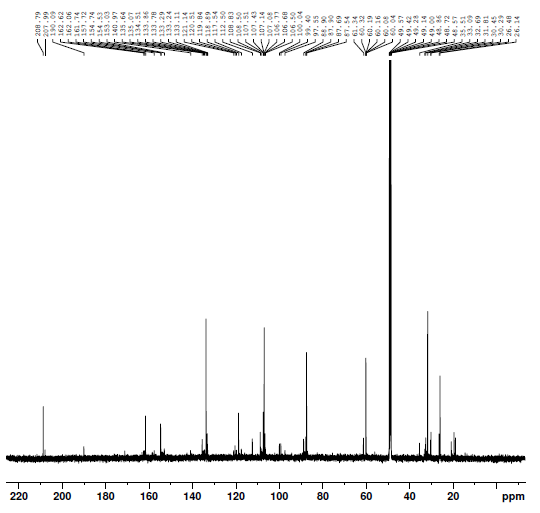


**S26 Fig. ^13^C-NMR spectrum (150 MHz, CD_3_OD) of 13**


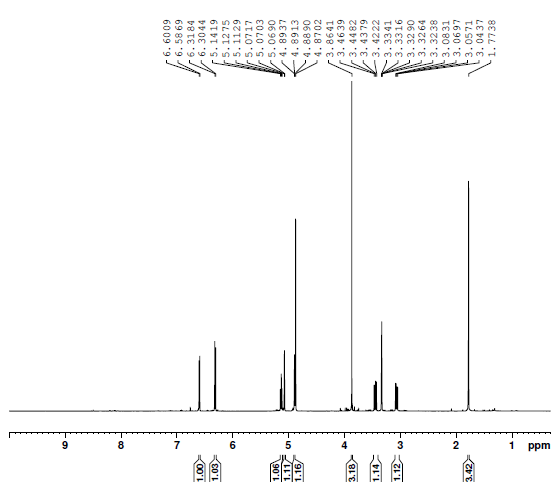


**S27 Fig. ^1^H-NMR spectrum (600 MHz, CD_3_OD) of 14**


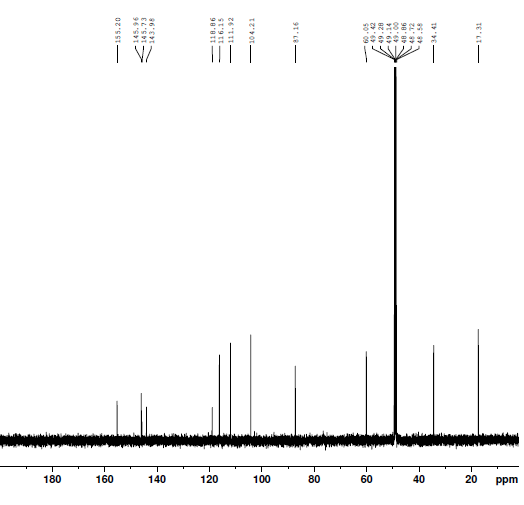


**S28 Fig. ^13^C-NMR spectrum (150 MHz, CD_3_OD) of 14**


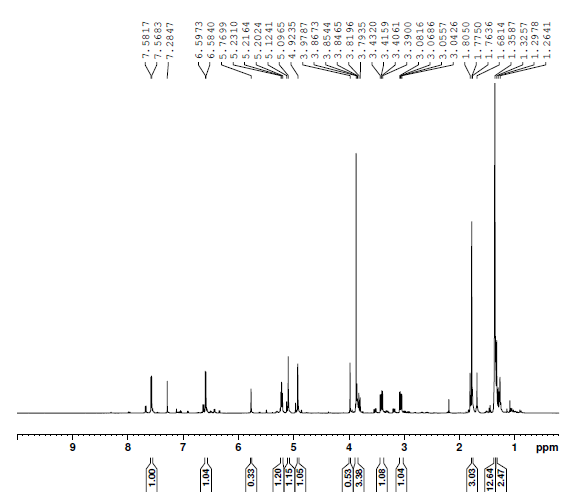


**S29 Fig. ^1^H-NMR spectrum (600 MHz, CDCl_3_) of 15**


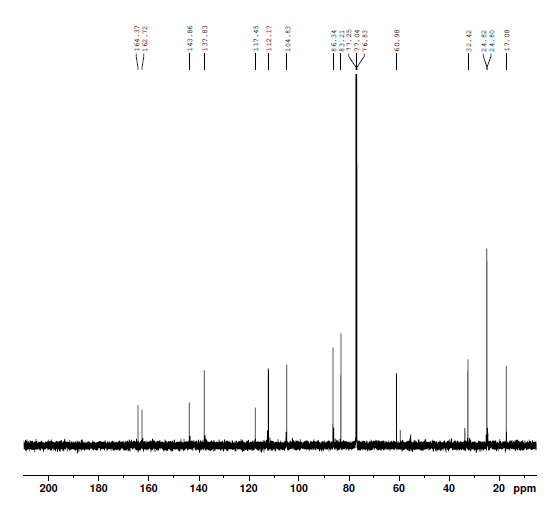


**S30 Fig. ^13^C-NMR spectrum (150 MHz, CDCl_3_) of 15**


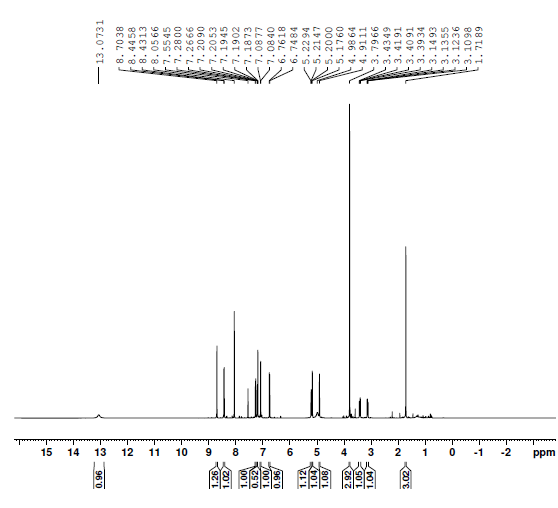


**S31 Fig. ^1^H-NMR spectrum (600 MHz, Pyridine-*d_5_*) of 16**


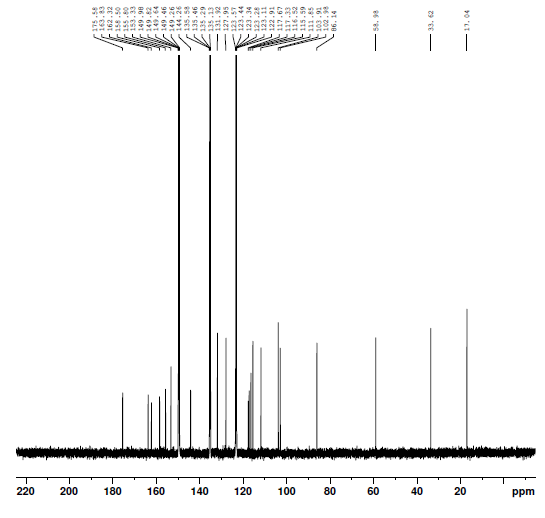


**S32 Fig. ^13^C-NMR spectrum (150 MHz, Pyridine-*d_5_*) of 16**


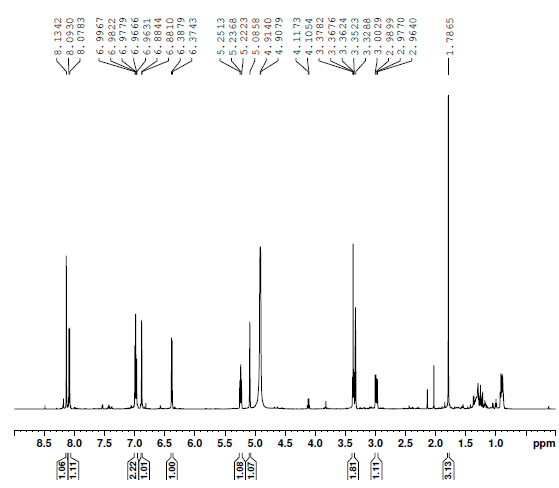


**S33 Fig. ^1^H-NMR spectrum (600 MHz, CD_3_OD) of 17.**


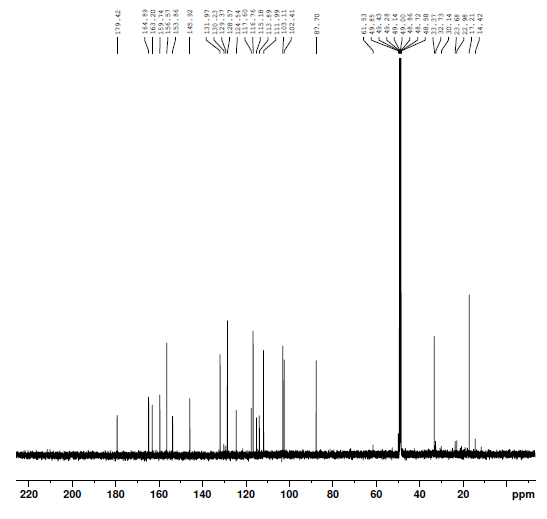


**S34 Fig. ^13^C-NMR spectrum (150 MHz, CD_3_OD) of 17**


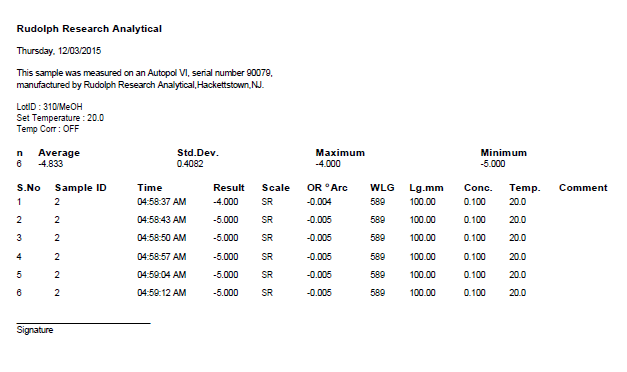


**S35 Fig. Ord result of 9a**


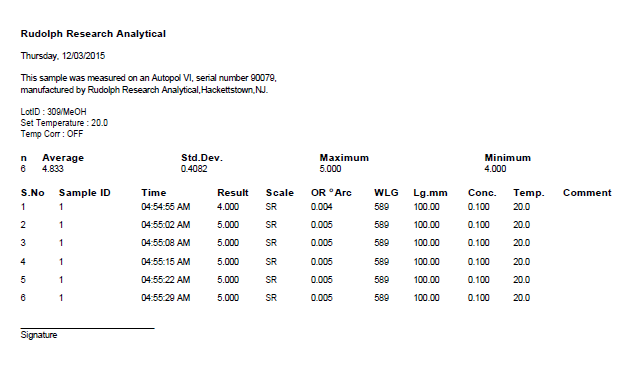


**S36 Fig. Ord result of 9b**
